# Diaphragm assessment by two dimensional speckle tracking imaging in normal subjects

**DOI:** 10.1186/s12871-016-0201-6

**Published:** 2016-07-25

**Authors:** Sam R. Orde, Andrea J. Boon, Daniel G. Firth, Hector R. Villarraga, Hiroshi Sekiguchi

**Affiliations:** 1 Division of Cardiovascular Diseases, Mayo Clinic, Rochester, Minnesota USA; 2 Department of Physical Medicine and Rehabilitation/Department of Neurology, Mayo Clinic, Rochester, Minnesota USA; 3 Department of Family Medicine, Mayo Clinic, Rochester, Minnesota USA; 4 Division of Pulmonary and Critical Care Medicine, Mayo Clinic, Rochester, Minnesota USA; 5 Department of Intensive Care, Nepean Hospital, Sydney, Australia

**Keywords:** Diaphragm, Ultrasound, Speckle tracking

## Abstract

**Background:**

Conventionally, ultrasonographic assessment of diaphragm contractility has involved measuring respiratory changes in diaphragm thickness (thickening fraction) using B-mode or caudal displacement with M-mode. Two-dimensional speckle-tracking has been increasingly used to assess muscle deformation (‘strain’) in echocardiography. We sought to determine in a pilot study if this technology could be utilized to analyze diaphragmatic contraction.

**Methods:**

Fifty healthy adult volunteers with normal exercise capacity underwent ultrasound imaging. A linear array transducer was used for the assessment of diaphragm thickness, thickening fraction (TF), and strain in the right anterior axillary line at approximately the ninth intercostal space. A phased array transducer was applied subcostally for the assessment of diaphragm displacement on the right mid-clavicular line. Diaphragmatic images were recorded from the end of expiration through the end of inspiration at 60 % maximal inspiratory capacity. Diaphragm strain was analyzed off-line by speckle tracking imaging. Blinded inter- and intra-rater variability was tested in 10 cases.

**Results:**

Mean right diaphragm thickness at end-expiration (±SD: standard deviation) was 0.24 cm (±0.1), with TF of 45.1 % (±12) at 60 % peak inspiratory effort. Mean right diaphragm caudal displacement was 4.9 cm (±1). Mean right diaphragm strain was -40.3 % (±9). A moderate correlation was seen between longitudinal strain and TF (R^2^ 0.44, *p* < 0.0001). A weak correlation was seen between strain and caudal displacement (R^2^ 0.14, *p* < 0.01), and an even weaker correlation was seen between caudal displacement and TF (R^2^ 0.1, *p* = 0.04). Age, gender, and body mass index were not significantly associated with right diaphragm strain or TF. Although inter- and intra-rater variability was overall good for TF, caudal displacement, and strain (inter-rater R^2^; 0.8, 0.9, and 0.7, respectively [*p* < 0.01], intra-rater R^2^; 0.9, 0.7, and 0.9, respectively [*p* < 0.01]), strain values did have a slightly lower inter-rater repeatability.

**Conclusions:**

Diaphragmatic strain estimated by speckle tracking imaging was associated with conventional ultrasound measures of diaphragmatic function (TF and caudal displacement). Further clinical studies are warranted to investigate its clinical utility.

**Electronic supplementary material:**

The online version of this article (doi:10.1186/s12871-016-0201-6) contains supplementary material, which is available to authorized users.

## Background

**Fig. 1:**
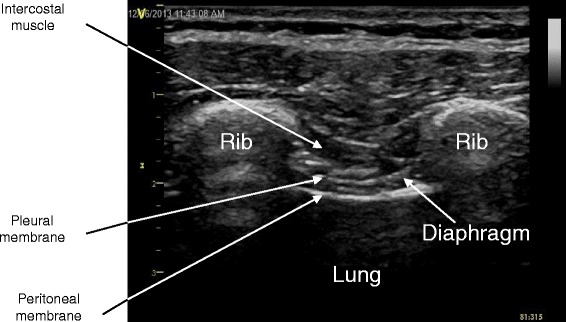
Ultrasound image of normal diaphragm in zone of apposition. A linear array transducer was applied with the use of M-mode ultrasound at the right anterior axillary line at approximately the ninth intercostal space

**Fig. 2:**
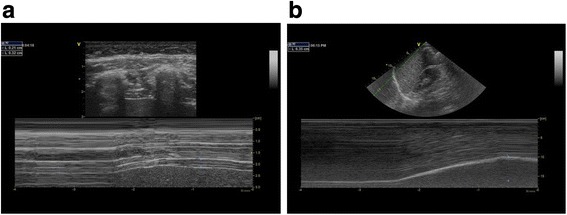
Examples of conventional assessment of diaphragm function. **a** Diaphragm thickening Fraction = diaphragm diameter at end-inspiration minus diaphragm diameter at end-expiration divided by diaphragm diameter at end-expiration, expressed as a percentage: [(DDinsp – DDexp)/DDexp] x 100. **b** Diaphragm caudal displacement with inspiration measured with a phased array transducer with the use of M-mode ultrasound in mid-axillary line in subcostal position

Measurement of transdiaphragmatic pressures has been considered the gold standard for the assessment of diaphragmatic function; however, it is rarely conducted due to its invasiveness [1]. While several alternative modalities have been used, such as chest radiography, video fluoroscopy, pulmonary function testing, electromyography, and nerve conduction studies, their accuracy and reproducibility may be modest and some are invasive or cause radiation exposure [2]. Recently, there has been growing interest in ultrasonographic assessment of the diaphragm due to its portability, lack of radiation exposure, non-invasiveness, and ease of repeatability. Previous studies have demonstrated the utility of diaphragm thickening fraction (TF) and caudal displacement with inspiration as markers of diaphragmatic function, measured via M-mode in the zone of diaphragm apposition and via two-dimensional (2D) B-mode imaging in the subcostal area, respectively [3–6] (Figs. 1 and 2). Measurements have been shown to be feasible, reproducible, and accurate [7–9]. Other studies have shown that a large variety of disorders can result in diaphragm thinning and dysfunction, which was associated with respiratory complications and prolonged weaning from mechanical ventilation [10, 11].

**Fig. 3:**
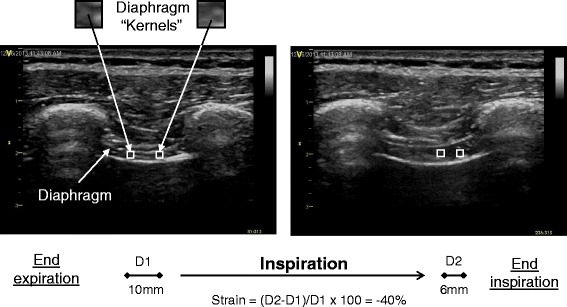
Representation of diaphragm strain assessment. D1 = Distance between diaphragm ‘kernels’ (unique groups of grey-scale pixels) at end-expiration. D2 = Distance between diaphragm ‘kernels’ at end-inspiration

Measuring diaphragm thickening and displacement are indirect estimates of diaphragm muscle contraction as they do not assess ‘longitudinal’ muscle shortening, ie: in the ‘plane’ of muscle fiber motion. Speckle tracking analysis has the potential to describe this function. Speckle tracking is a novel analysis method used in echocardiography to determine myocardial fiber deformation [12]. Ultrasound images are made up of different grey-scale pixels, and the speckle tracking software follows unique groups of these pixels (known as ‘kernels’) and measures their displacement and how different ‘kernels’ move in relation to one another (deformation). The degree of deformation is known as ‘strain’ and negative values indicate ‘kernels’ coming closer together. For example, a strain value of -40 % indicates local muscle fiber shortening of 40 %. The more negative a number, the greater the degree of deformation and the greater the contraction (Fig. 3).

In this pilot study, we sought to determine (1) the feasibility of speckle tracking in assessing diaphragm function, (2) how this compares to conventional methods of diaphragm function assessment by ultrasound: TF and caudal displacement, and (3) reproducibility of speckle tracking analysis of the diaphragm.

## Methods

Fifty adult subjects were included in the study. Inclusion criteria comprised of healthy adults at age over 18 years with a normal exercise tolerance (defined as being able to climb three flights of stairs with relative ease). Exclusion criteria were previous history of neuromuscular disease, chronic respiratory issues (defined as moderate to severe restrictive or obstructive lung disease), and cardiopulmonary or abdominal surgery. Ultrasonography was conducted in subjects during their inspiratory efforts. Each inspiration effort was standardized by using an incentive spirometry device (Hudson RCI®, Voldyne 5000). Using a predictive normogram based on sex, age and height [13], peak inspiratory capacity was calculated for each individual. Study subjects were instructed to inspire to 60 % of their peak inspiratory capacity in one second. A 60 % inspiratory breath was chosen to limit descent of the lung into the image but to ensure adequate diaphragm movement. The study was approved by the Mayo Institutional Review Board (11-002744), and all subjects provided informed consent.

### Ultrasound imaging and analysis

Imaging was performed using a commercially available ultrasound machine (Vivid E9, General Electric Healthcare, Milwaukee, WI) with a linear array transducer (2.5-8 MHz) and a phased array transducer (1.6-6 MHz) by S.O.: Australian Intensive Care specialist, board certified in standard and advanced echocardiography in America (Fellow of American Society of Echocardiography, FASE) and Australia (Diploma Diagnostic Ultrasound, DDU). Subjects were positioned semi-recumbent (45° head-up). The linear array transducer was first placed perpendicular to the angle of the lower ribs, with the transducer marker directed cephalad. Images of two adjacent ribs were seen at approximately the same level with the diaphragm running parallel to the transducer surface (Fig. 1). Image acquisition began in the right mid-axillary line above the costal margin, where the zone of apposition of the diaphragm could be visualized at approximately the 9^th^ intercostal space (Additional file 1: Figure S1a). If the lung expansion obscured the optimal images during inspiration (‘lung curtain’ sign) [14], then the transducer was moved anteriorly along the rib space toward the anterior axillary line until no ‘lung curtain’ sign was seen (Additional file 1: Figure S1b). Only one focus level was chosen, and the depth was optimized in order to visualize the diaphragm as clearly as possible throughout inspiration with maximal frame rate. Attempts were made to enhance sonographic speckles in the diaphragm by slight transducer angulation.

M-mode imaging was conducted with the cursor line equidistant between the two ribs perpendicular to the diaphragm. Diaphragm thickness was measured, excluding the outer layers of pleura and peritoneum, from the inner-edge to the inner-edge at end-expiration and at end-inspiration (Fig. 2a). TF was calculated by: diaphragm diameter at end-inspiration minus diaphragm diameter at end-expiration divided by diaphragm diameter at end-expiration, expressed as a percentage: [(DDinsp – DDexp)/DDexp] x 100.

**Fig. 4:**
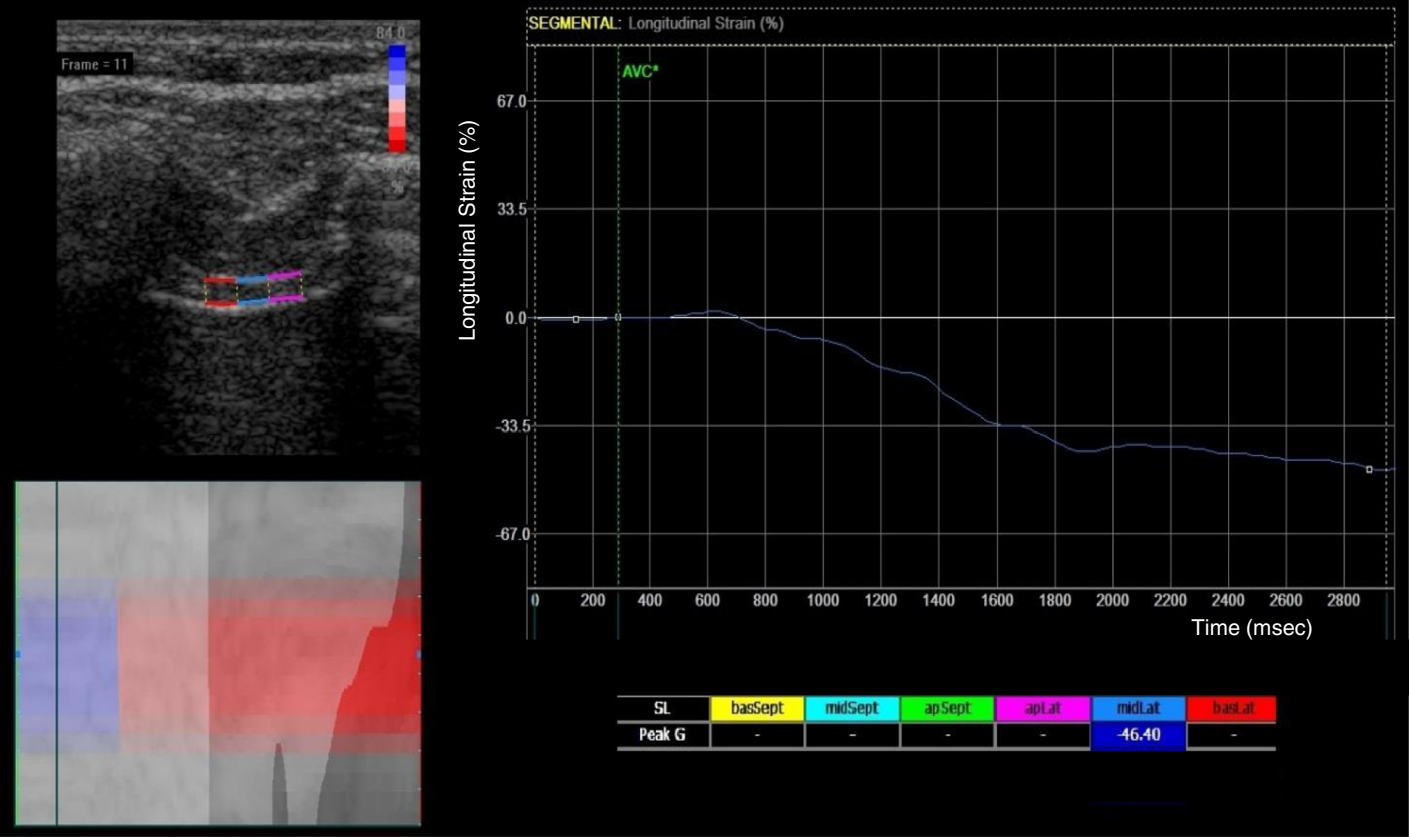
Longitudinal strain of diaphragm during inspiration. The x-axis represents time (millisecond), and the y-axis represents longitudinal strain (%). Strain is a measure of relative deformation and is a negative value. In this example, the central portion of the region of interest (depicted in blue in the ultrasound image at the upper-left corner) was traced and measured as – 46.4 % (displayed in the column below the x-axis). The more negative value means the higher degree of deformation (contraction)

Subsequently, three-second clips were recorded for off-line speckle-tracking analysis using EchoPacs (GE Healthcare, Milwaukee, MI). Various assumptions that EchoPacs requires for standard speckle-tracking were contradicted, for example: absent ECG tracings, use of linear array transducer, shallow depth, abnormal tracing, lack of drift compensation, and optimized spatial and temporal smoothing. Apical four chamber longitudinal analysis was used, and the inner surface of the two hyperechogenic lines bordering the diaphragm muscle (the pleura and peritoneal surface) were traced and followed during an inspiratory breath to contain the diaphragm muscle within the barriers. The central portion of the region of interest was analyzed only (Figs. 3 and 4, and Additional file 2: Video 1).

Diaphragm caudal displacement was calculated by imaging with a phased array transducer in the right anterior to mid-clavicular line with the transducer aimed dorso-cranially (Additional file 3: Figure S2). M-mode imaging was used with the cursor line placed at the dome of the diaphragm. Diaphragm displacement was calculated as the maximal height change of the echogenic line on the M-mode image (Fig. 2b).

In order to assess inter-rater variability, 10 subjects’ images were analyzed by two different observers (S.O. and D.F.) blinded to each other’s assessment. Intra-rater variability was evaluated in 10 subjects by performing the same imaging and analysis on a different examination date (by S.O.).

### Statistical analysis

Data analysis was performed using JMP software version 10.0.0 (SAS Institute Inc. North Carolina). Continuous variables were expressed as mean ± standard deviation (SD) or median ± interquartile range (IQR) if not normally distributed. Normality was tested by visual assessment and the Shapiro-Wilk test [15]. Non-normally distributed data (eg: right diaphragm longitudinal strain) underwent logarithmic transformation. Pearson’s correlation coefficient was used to assess correlation between continuous variables and t-test for direct comparison. *P* values <0.05 were considered statistically significant, and all probability values were 2 sided. Reproducibility was expressed by interclass correlation coefficient [16], Pearson’s correlation, and Bland-Altman’s coefficient of repeatability (twice the SD of differences in repeated measurements) [17, 18].

## Results

A total of 50 subjects were prospectively enrolled for the study. Twenty-eight (56 %) were female with a median age of 37 years old (IQR: 30.2 to 39.8). The median body mass index (BMI) was 22.8 kg/m^2^ (IQR 20.4–24.9). Estimated inspiratory capacity based on age, sex, and height had a median of 2900 ml (IQR 2375 to 3100). Baseline characteristics of the subjects by sex are described in Table 1.

**Table 1:** Baseline characteristics of 50 normal subjects

Variable (Mean ± SD)	Male (*N* = 22)	Female (*N* = 28)	*P*-value
Age (years)	37.2 ± 10	35.9 ± 7	0.61
BMI (kg/m^2^)	24.7 ± 2	21.3 ± 2	<0.001
60 % inspiratory capacity (cc)	1860 ± 128	1504 ± 204	<0.001

**Fig. 5:**
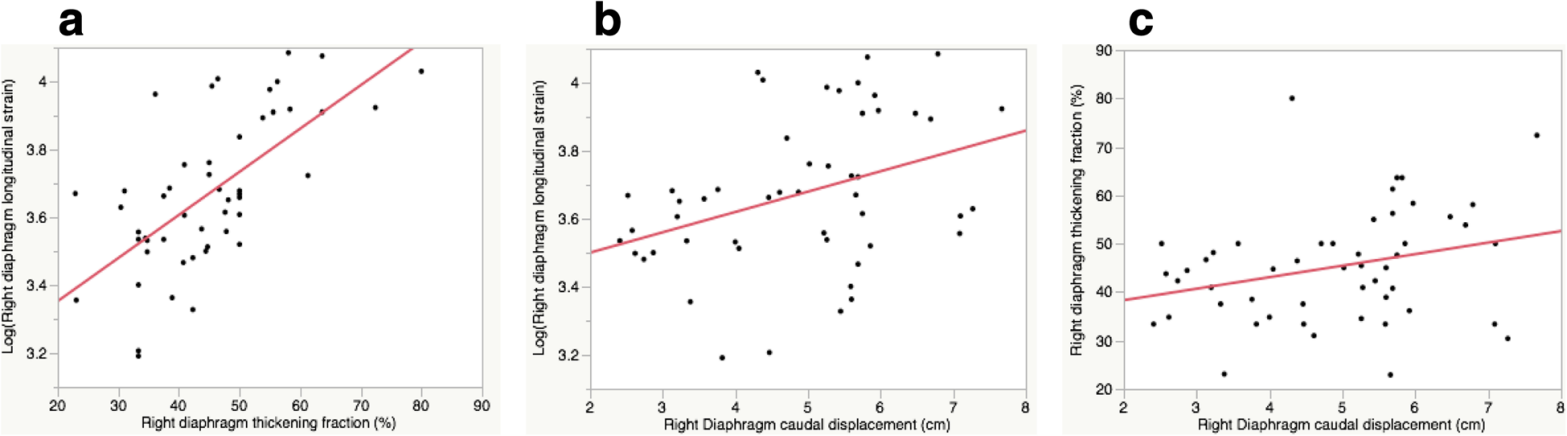
Graphical representation of correlation among various diaphragm assessment methods. **a** Log(Longitudinal strain) vs. thickening fraction (Log Strain = 3.1 – 0.01*Thickening fraction; R^2^ 0.44, p < 0.0001), **b** Log(Longitudinal strain) vs. caudal displacement (Log Strain = 3.4 + 0.6*Displacement; R^2^ 0.14, *p* < 0.01), and **c** Thickening fraction vs. caudal displacement (Thickening fraction = 33.5 + 2.4*Displacement; R^2^ 0.1, *p* = 0.04)

The mean diaphragm thickness at end-expiration, TF, caudal displacement, and longitudinal strain values are listed in Table 2. The mean right diaphragmatic thickness was 0.24 cm (±0.1) and 0.35 cm (±0.1) at the end expiration and inspiration, respectively. The mean TF was 45.1 % (±12). The mean caudal displacement was 4.9 cm (±1). The right diaphragmatic longitudinal strain was -40.3 % (±9). No sex-specific differences were seen in these values. Neither BMI nor estimated inspiratory capacity demonstrated a significant correlation with diaphragmatic thickness, TF, caudal displacement, or strain on the univariate linear regression analysis. There was a moderate association between right diaphragm longitudinal strain (logarithmic scale) and TF (R^2^ 0.44, *p* < 0.0001). Although a weaker association with right diaphragm strain and caudal displacement was observed (R^2^ 0.14, *p* < 0.01), its visual correlation was not as strong as one between TF and caudal displacement (R^2^ 0.1, *p* < 0.0001) (see Fig. 5).

**Table 2:** Ultrasound values of right diaphragm analysis

Variable (Mean ± SD)	All subjects	Male	Female	*P*-value
Diaphragm thickness end expiration (cm)	0.24 ± 0.1	0.25 ± 0.1	0.24 ± 0.1	0.94
Thickening fraction (%)	45.1 ± 12	42.6 ± 10	47.1 ± 13	0.17
Caudal displacement (cm)	4.9 ± 1	4.6 ± 1.4	5.1 ± 1.4	0.9
Strain (%)	−40.3 ± 9	−39.5 ± 8	−40.9 ± 10	0.58

Inter-and intra-operator reliability was good (Table 3). The correlation between the measurements obtained by two operators was highly significant in the right diaphragm TF (ICC 0.9, R^2^ 0.8, *p* < 0.001, coefficient of repeatability 2.6 % and 8 % respectively) as well as caudal displacement (ICC 0.9, R^2^ 0.9, *p* < 0.001, coefficient of repeatability 14.5 %). The correlation in the right diaphragm longitudinal strain measurements was also significant (ICC 0.9, R^2^ 0.7, *p* = 0.004), with a slightly higher coefficient of repeatability (24.3 %). A similar trend was seen in intra-operator variability. The correlation in measurements of TF, caudal displacement, and strain were highly significant (R^2^ 0.94, 0.72, and 0.91, respectively).

**Table 3:** Reproducibility: inter- and intra-rater reliability

Variable	ICC (95 % CI)	R^2^	R^2^ *p*-value	Coefficient of repeatability (%)
Inter-rater				
Thickening fraction	0.95 (0.78–0.99)	0.80	<0.001	8.0
Caudal displacement	0.97 (0.79–0.98)	0.90	<0.001	14.5
Longitudinal strain	0.90 (0.61–0.98)	0.70	0.004	24.3
Intra-rater				
Thickening fraction	0.98 (0.83–0.99)	0.94	<0.001	14.9
Caudal displacement	0.88 (0.47–0.97)	0.72	<0.002	33.6
Longitudinal strain	0.96 (0.88–0.99)	0.91	<0.001	19.4

Abbreviations: ICC, Interclass correlation coefficient; 95 % CI, 95 % confidence interval, R^2^, Pearson’s correlation coefficient

## Discussion

Our study demonstrated that diaphragmatic assessment using speckle tracking with a linear array transducer was feasible and reproducible, albeit more variable than conventional assessment by TF with use of M-mode ultrasonography. This supports a recent study that reported 2D speckle tracking assessment using a low frequency phased array transducer placed on the right subcostal, mid-clavicular line [19]. In this study, longitudinal muscle contraction of the diaphragm was estimated by assessing the entire dome of the diaphragm, whereas we examined the diaphragm in the zone of apposition with use of a high frequency linear array transducer. We found a mean right diaphragm longitudinal strain value of -40.3 % ±9 at a 60 % maximal inspiratory capacity. This corresponded with a mean TF of 45.1 % (±12), which was in keeping with data from a larger population of normal subjects [6]. Mean right diaphragm thickness at end-expiration in our study was 0.24 mm ±0.1 and caudal displacement 4.9 cm (±1), which are also comparable to previous literature [6, 20].

Although there was a moderate correlation seen between right diaphragm longitudinal strain and TF, the correlation between strain and caudal displacement was weak. The significance of correlation, albeit not a strong one, suggests that at least speckle tracking may be reasonable method for analysing diaphragm function. We have to be mindful of the fact that strain and TF are measuring function in different planes and direction: for example diaphragm longitudinal strain could be considered to measure deformation in the ‘x plane’, while TF is a measure of deformation in the ‘y plane’. However, a modest correlation between strain and TF can be explained through the principle of conservation of volume, as previously proposed in the assessment of incompressible tissue [21]. Recent studies suggested that diaphragm displacement is not as representative of respiratory effort or diaphragmatic function as diaphragm thickening [22, 23]. There was no strong correlation found between diaphragm displacement and indices of respiratory workload, nor was between displacement and TF [22]. Interestingly, in our study, a poor correlation was seen between strain and caudal displacement.

A significant difference was seen between male and female subjects for BMI and 60 % inspiratory capacity; however, this difference was not seen with respect to diaphragm function. Our result may represent the hypothesis that not the absolute value of inspiratory capacity, but the proportion (or percentage, here 60 %) of the maximal inspiratory capacity is an important factor to dictate the needed diaphragmatic strength and contraction when adjusted by sex and body size.

The diaphragm acts as a primary muscle of ventilation, and its dysfunction can result in dyspnoea and complications such as difficultly in liberation from mechanical ventilation [11], yet it is not routinely evaluated or monitored [24]. Ultrasound has been recognized as a new tool to examine the diaphragm non-invasively in various clinical settings [25, 26]. However, conventional methods, such as TF, or caudal displacement assessed by M-mode have limitations, such as angle dependence and translational error (where other areas of diaphragm move into the ‘line of sight’). 2D speckle tracking follows specific grey-scale pixels (known as ‘kernels’) in specified regions of interest and is less prone to the errors of traditional ultrasound methods as it is relatively angle independent. Also tracking can help differentiate passive movement from active contraction as the ‘kernels’ do not move closer together in passive motion, whereas they do with any active contraction [27]. Our study may help to provide a novel quantifiable method of diaphragm function analysis: longitudinal strain of the diaphragm assessed by speckle tracking.

There are limitations to our study. Primarily, the reference ultrasound methods used in this study for measuring diaphragm function have not been validated in a large study or in a study using trans-diaphragmatic pressure as a gold standard [3, 28]. However, diaphragm TF and caudal displacement have been shown to be relevant in the clinical environment and are commonly used non-invasive tools [2]. Secondly, the technology used in our study is not designed for diaphragm analysis. Several assumptions that the software makes which help it to be sensitive in determining myocardial deformation needed to be broken. Third, the speckle tracking software automatically generated three regions of interest when the diaphragm was traced. We only analysed the central region of interest as the peripheral sections did not have appropriate tracking. Fourth, the time taken to perform analysis was considerable relative to a clinical situation. This limits the current clinical utility of this method of assessment. However, as technology improves, speckle tracking may become easier and has the potential to add to non-invasive diaphragm function assessment. Finally, our pilot study enrolled a small number of patients and did not include subjects with abnormal diaphragmatic function. Sample size was difficult to statistically estimate due to lack of prior data. Further studies enrolling subjects with abnormal diaphragmatic function and/or critical illness, with comparison to electromyography or transdiaphragmatic pressure measurements may be warranted.

## Conclusion

2D speckle tracking analysis of the right diaphragm is feasible and reproducible. It is associated with conventional ultrasound measures of diaphragmatic function (TF and caudal displacement). It provides unique information on longitudinal muscle deformation (strain) which conventional methods of ultrasound analysis, such as M-mode, are unable to provide. Although this form of analysis can take considerable time to perform, technological progress may help make this a useful clinical tool in the future.

## Abbreviations

2D, Two dimensional; DDexp, Diaphragm diameter end-expiration; DDinsp, Diaphragm diameter end-inspiration; ICU, Intensive Care Unit; IQR, Interquartile range; SD, Standard deviation; TF, Thickening fraction.

## Additional files

Additional file 1: Figure S1. Linear array transducer application for the assessment of diaphragm thickness and strain. (a) Image acquisition in the right mid-axillary line above the costal margin, where the zone of apposition of the diaphragm can be visualized at approximately the 9^th^ intercostal space. (b) If the lung expansion obscures the optimal images during inspiration (‘lung curtain’ sign), then the transducer can be moved anteriorly along the intercostal space toward the anterior axillary line until no ‘lung curtain’ sign is seen. (PPTX 1272 kb)

Additional file 2: Video 1. Speckle-tracking analysis of the diaphragm with use of a linear array transducer. The inner surface of the two hyperechogenic lines bordering the diaphragm muscle were traced and followed during an inspiratory breath. The central portion of the region of interest (depicted in blue) was analyzed. (7.63 MB)

Additional file 3: Figure S2. Phased array transducer application for the assessment of diaphragm displacement. Diaphragm caudal displacement can be measured by imaging with a phased array transducer in the right anterior to mid-clavicular line with the transducer aimed dorso-cranially. (PPTX 648 kb)
