# Supplementary figures and images for: Diaphragm assessment by two dimensional speckle tracking imaging in normal subjects
Source: BMC Anesthesiol. 2016 Jul 25;16:43. doi: 10.1186/s12871-016-0201-6 (PMC4960718; doi:10.1186/s12871-016-0201-6)

## Slide 1
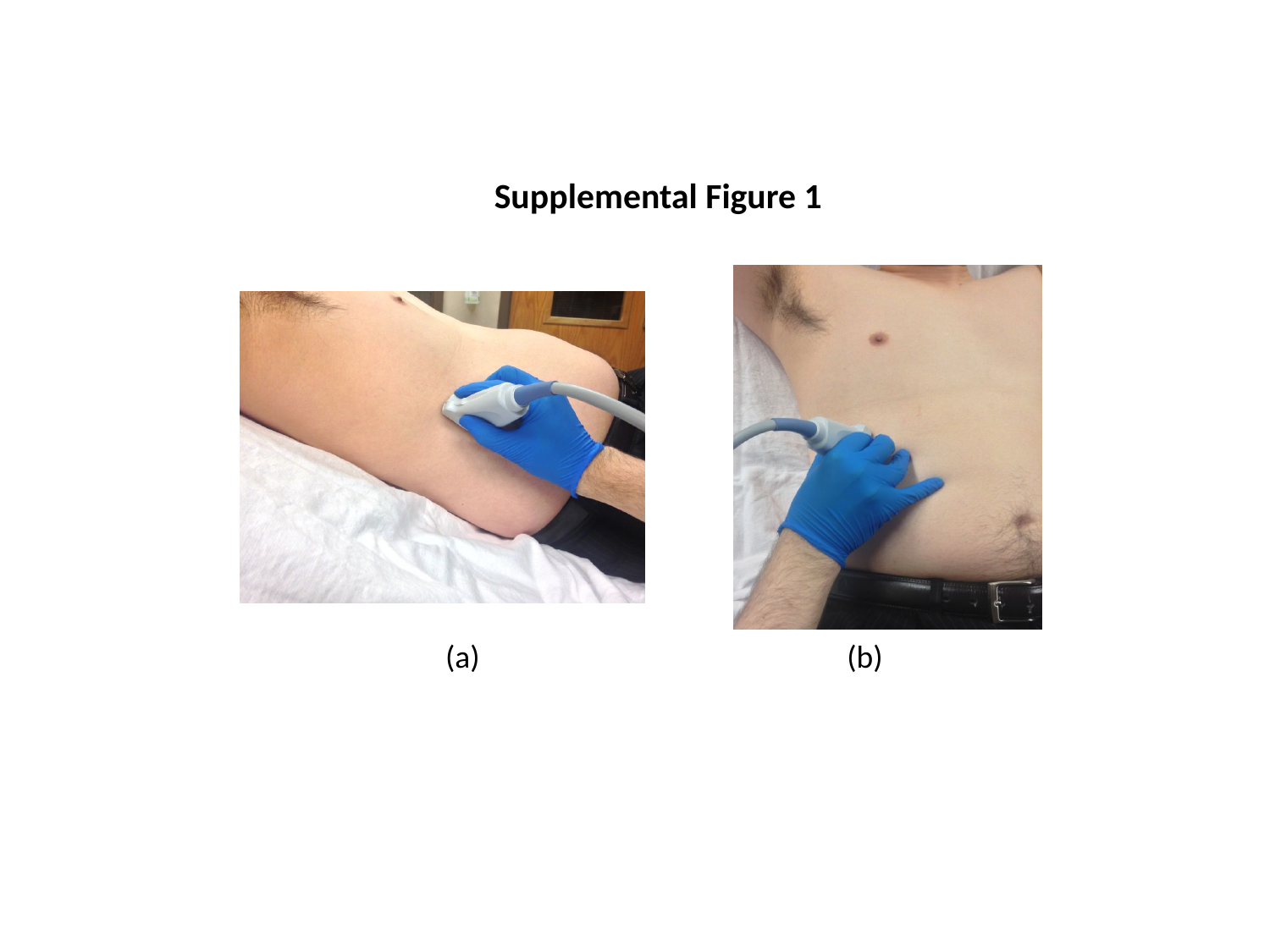

Supplemental Figure 1
(a)
(b)

Supplement: Supplementary file 1 — Linear array transducer application for the assessment of diaphragm thickness and strain. (a) Image acquisition in the right mid-axillary line above the costal margin, where the zone of apposition of the diaphragm can be visualized at approximately the 9th intercostal space. (b) If the lung expansion obscures the optimal images during inspiration (‘lung curtain’ sign), then the transducer can be moved anteriorly along the intercostal space toward the anterior axillary line until no ‘lung curtain’ sign is seen. (PPTX 1272 kb) [file 12871_2016_201_MOESM1_ESM.pptx]

## Slide 1
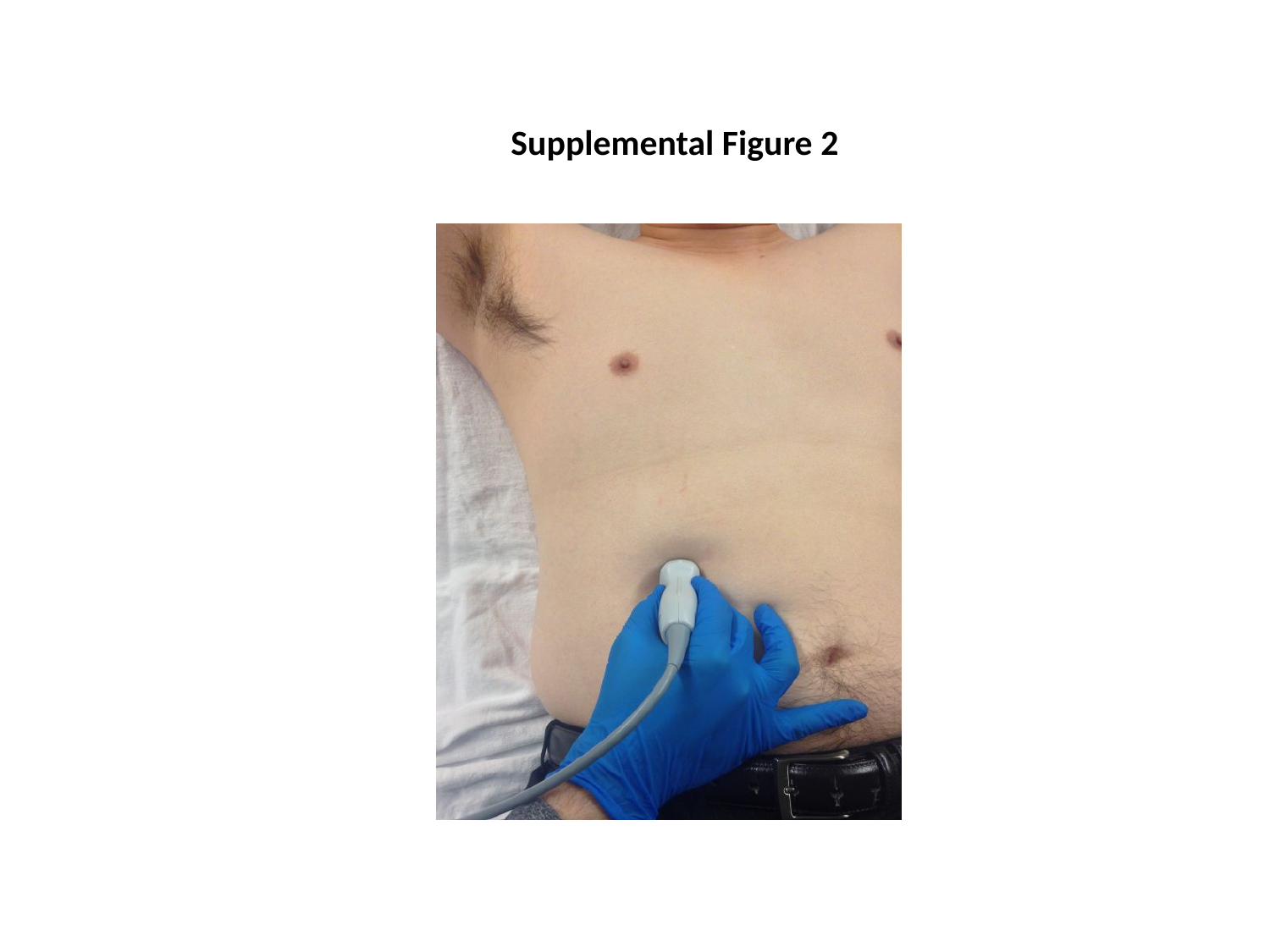

Supplemental Figure 2

Supplement: Supplementary file 3 — Phased array transducer application for the assessment of diaphragm displacement. Diaphragm caudal displacement can be measured by imaging with a phased array transducer in the right anterior to mid-clavicular line with the transducer aimed dorso-cranially. (PPTX 648 kb) [file 12871_2016_201_MOESM3_ESM.pptx]
